# Hydration and Fortification of Common Bean (*Phaseolus vulgaris* L.) with Grape Skin Phenolics—Effects of Ultrasound Application and Heating

**DOI:** 10.3390/antiox13050615

**Published:** 2024-05-18

**Authors:** Gloria Bonassi, Vera Lavelli

**Affiliations:** Department of Food, Environmental and Nutritional Sciences (DeFENS), University of Milan, 20133 Milan, Italy; gloria.bonassi@studenti.unimi.it

**Keywords:** legume, hydration, heating, flavonol, flavanol, phenolic acids, anthocyanin

## Abstract

Ultrasound (US)-assisted soaking combined with fortification with red grape skin (GS) phenolics was applied on two *Phaseolus* varieties, namely White Kidney Bean (WKB) and Cranberry Bean (CB), before heat treatment. The aims were to investigate: (a) the effect of US application on the kinetic of hydration; (b) the extent of absorption of different phenolic classes of GS into the beans and the resulting effect on antioxidant activity; (c) the effects of heat treatment on the phenolic fraction and antioxidant activity of GS extract- and water-soaked beans. US fastened the soaking step of both WKB and CB beans, which showed the sigmoidal and the downward concave shape hydration curves, respectively. Anthocyanins, flavonols, flavanol and phenolic acids levels increased with GS soaking, but US application was effective only for increasing the level of flavonols, while it favored the loss of endogenous phenolic acids and it did not affect the uptake of anthocyanins and flavanols. Heat treatment decreased the levels of most of phenolic compounds, but increased the levels of monomeric flavanols. Overall, the antioxidant activity was 40% higher in WKB and 53% higher in CB upon GS-fortification than in the control beans, despite the effects of heating. This fortification strategy could be applied for value addition of varieties low in phenolics or as a pre-treatment before intensive processing.

## 1. Introduction

Crops belonging to the *Leguminosae* family are particularly interesting as a protein source and for their capability to fix atmospheric nitrogen, which makes them independent of fuel-driven nitrogen fertilizers [1,2]. The antioxidant content of legumes is also of interest for their potential bioactivity, although it is strongly dependent on the species [3,4]. Among the plant family *Leguminosae*, common bean (*Phaseolus vulgaris* L.) is the most consumed [1,5]. According to the FAO, common bean production has increased from 25.1 to 28.3 million tons in the decade 2012–2022. Asia shares 50% of the global production of common beans, and Myanmar, India, Brazil, China, America were the top five dry-bean-producing countries in the world in the last ten years [5].

Traditionally, dry legume seeds need to be soaked before further processing. Indeed, the raw seeds absorb water during soaking, allowing for the removal of anti-nutritional factors and faster cooking. The grain hydration is associated with a relevant loss of water-soluble antioxidants [6]. Moreover, soaking is a slow and batch process and, hence, strategies have been studied to accelerate water uptake. Increasing the soaking temperature lowers water viscosity which, in turn, increases the water diffusivity via enlarged bean pores, thus increasing the water uptake rate. However, soaking at high temperatures results in thermal degradation of bioactive compounds and decreases equilibrium moisture content [7]. More recently, pulsed electric fields (PEF) have been applied to accelerate the soaking process at 4 kV/cm, frequency = 2 Hz, pulse width 15 μs, pulse numbers from 200 to 1000 for 400 min [8]. PEF application during soaking increases the temperature of the hydration medium and promotes electroporation [8]. Alternatively, the use of ultrasounds (US) at frequencies in the range from 25 [9] to 80 kHz [10], volumetric power in the range from 17 [11] to 7.5 W/L [12] and processing time up to 360 min was proposed to decrease soaking time. The application of US enhances the mass transfer during hydration process by two main mechanisms. The first direct effect of US is related to the ultrasonic wave traveling through the food matrix, which causes the alternative compression and expansion of the medium, facilitating the water entrance into the grain pores by pumping [13]. Further, this mechanism also causes the compression and expansion of the tissues, which behave as a sponge, squeezing water [14]. The second indirect effect of US is related to structural modifications due to the acoustic cavitation, which causes the cell and tissue disruption. This mechanism forms microchannels inside the beans, thereby improving the mass transfer and increasing the equilibrium moisture content [15,16].

US-based strategies that accelerate water transfer during soaking can also be extended to fortify the food matrix. Considering staple starch-based foods, US-fortification was extensively studied for potatoes and rice. In potatoes, US pretreatment prior to vacuum impregnation improves matrix permeability, as assessed by image analysis, and was applied to achieve high iron impregnation [17]. In a further study, the approach was extended by combining the incorporation of iron with that of a small organic molecule, namely ascorbic acid [18]. In rice, US application results in the formation of a highly porous surface, as proven by X-ray computed tomography, which is suitable for incorporation of guest molecules [19]. This finding has opened up a new way for rice fortification with vitamin B5 [20], iron [19] and folic acid [21]. The approach of US-fortification was introduced in legume processing by using a valuable micronutrient, namely, ferrous sulphate dissolved in the soaking solution of beans to increase iron content in the final product [15].

Strategies that accelerate water transfer during soaking and, thus, effectively decrease processing time, also result in high loss of nutrients, such as phenolic compounds [8]. Hence, in the current study, bean fortification with phenolics was investigated. One point to consider is that the liquid-to-solid ratio in the soaking process of bean needs to be high to successfully remove anti-nutritional factors by dilution. Hence, only a minor part of the soaking water enters the beans. For instance, in the previously mentioned study, a solid-to-liquid ratio of 10: 250 (*w*/*v*) was used and an average of 4% of the soaking solution was absorbed by beans [15]. The left-over soaking solution cannot be recycled because it contains antinutritional factors. Hence, a suitable soaking solution for fortification purpose should have high nutritional value and low cost. Moreover, the thermal stability of the incorporated phenolics in the bean matrix need to be verified to assess the residual amount of these compounds after cooking.

In this research, the use of a phenolic-rich soaking solution for bean hydration was studied for the first time, and the effects on phenolic levels and antioxidant activity in beans after US-assisted soaking and heating were investigated. The phenolic-rich extract obtained from grape skins (GS), a residue of winemaking, was used as a soaking water for hydration of two *Phaseouls* varieties, since it meets both the requisites of high nutritional value and low cost. In particular, the aims of study were to assess: (a) the effect of US application on the kinetic of hydration with either water or GS extract as a soaking solution; (b) the extent of absorption of different phenolic classes of GS into the beans and the resulting effect on antioxidant activity; (c) the effect of heat treatment (simulating cooking conditions) on the phenolic fraction and antioxidant activity of GS extract- and water-soaked beans.

## 2. Materials and Methods

### 2.1. Raw Materials

Two commercial grain varieties belonging to *Phaseolus vulgaris* L., namely white kidney bean (WHB) and cranberry bean (CB), were obtained from a local market. Red grape (*Vitis vinifera* L.) pomace (Barbera variety) was kindly provided by a winery located in Northern Italy. At the winery, the pomace was sieved (with a 5 mm sieve) to separate the skins from the seeds and frozen to inhibit microbial growth. Grape skins (GS) were transported frozen to the lab and dried at 50 °C for about 8 h using a Ignis model AKS201/IX/01 ventilated oven (Whirpool, Milan, Italy) before milling as described in the following paragraph. All standard compounds and chemicals were purchased from Sigma–Aldrich (Milan, Italy).

### 2.2. Extraction of Water-Soluble Grape Skin Phenolics

Dried GS were milled and sieved by using the Octagon Digital sieve shaker (Endecotts Ltd., London, UK), with certified sieves to obtain the fraction with particle sizes in the range 250–500 μm. Water-soluble phenolic extract was obtained by extraction of 20 g of dried GS powder with 500 mL of water with continuous stirring for 6 h at room temperature. The GS extract was recovered by filtration. The pH of the extract was measured with a model 62 pH meter (Radiometer, Copenhagen, Denmark). The extract was kept at 4 °C until use.

### 2.3. Moisture Content

Moisture content of the commercial dry beans was determined in triplicate by drying in a vacuum oven at 70 °C and 70 Torr for 16 h. Moisture content of beans during hydration and after heat treatment was obtained by mass balance, considering the increase in mass during processing and the initial moisture content. Moisture content was expressed as grams of water in 100 g of dry solids (d.s.).

### 2.4. Hydration and Heat Treatment of Beans

The hydration and heat-treatment processes were carried out in duplicate. To perform the hydration process, a previously proposed procedure was followed [16]. In brief, approximately 10 g of grains were placed inside a beaker with either 200 mL of distilled water or 200 mL of GS extract and submitted to US at 25 kHz with a volumetric power of 125 kW/L, using a model VCX 500 US sonicator (Ghiaroni & C, Milan, Italy). The temperature was maintained in the interval of 20–30 °C using an ice bath. In parallel, hydration was performed without US application at 20 °C. During the hydration process, at 15 min or 30 min intervals, the samples were drained, superficially dried and weighted. The process was stopped when a constant mass was reached (360 min). The hydrated beans were added to water at 1: 1.75 (*w*/*w*) ratio, placed in sealed glass bottles and heat-treated at 100 °C for 90 min, which represents an intensive heat-treatment, since cooking is generally performed for 30–90 min at 100 °C [22]. Heat-treated beans were cooled and grounded in the cooking water, then stored at 4 °C.

### 2.5. Mathematical Modeling

Hydration kinetics data of grains were fitted using the sigmoidal equation of Kaptso et al. [23] (Equation (1)) and the DCS equation of Peleg [24] (Equation (2)), as follows:

(1) $$Mt=M\infty /(1+\exp\left[-k*\left(t-\tau \right)]\right)$$

where *t* is time (min), *Mt* is the moisture content at time *t* (g_water_/100 g_d.s._), *M*_∞_ is the equilibrium moisture content (g_water_/100 g_d.s._), *τ* (min) describes the necessary time to reach the inflection point of the curve, and *k* (min)^−1^ is the water absorption rate; and

(2) Mt=Mo+t/(k1+k2∗t),

where *t* is time (min), *Mt* is the moisture content at time *t* (g_water_/100 g_d.s._), *Mo* is the initial moisture content (g_water_/100 g_d.s._), *k*1 (min·g_water_/100 g_d.s._)^−1^ and *k*2 (g_water_/100 g_d.s._)^−1^ are the Peleg’s kinetic parameters.

The correctness of fit was evaluated by the determination coefficient (R^2^), the root-mean-square deviation values (RMSD) and the normalized RMSD (NRSMD). 

### 2.6. Phenolic Extraction

Extraction of phenolic compounds was performed in triplicate on dry WKB and CB beans and WKB and CB beans submitted to soaking with water (H_2_O-S), soaking with GS extract (GS-S), US-assisted soaking with GS extract (GS-US-S), soaking with water and heating (H_2_O-S-H); soaking with GS extract and heating (GS-S-H), and US-assisted soaking with GS extract and heating (GS-US-H).

A procedure previously proposed in the literature was followed [25]. In brief, samples were milled with a Waring Commercial Blender (Fisher Scientific, Segrate, Italy) for 6 min at low speed. Approximately 0.5 g of sample was extracted with 7.5 mL of methanol:water:HCl (70:30:0.1, *v*/*v*/*v*), for 2 h at room temperature with continuous stirring. The mixture was centrifuged at 10,000× *g* for 6 min, the supernatant was recovered and the solid residue was re-extracted using 2.5 mL of the same solvent. The supernatants were pooled and kept at −20 °C until use.

### 2.7. Phenolic Analysis by HPLC 

The phenolic contents of GS and bean extracts were analyzed in duplicate as described previously [26], using a model Shimadzu LC-20 AD pump coupled to a model Shimadzu SPD-M20A photodiode array detector (DAD) and an RF-20 AXS operated by Labsolution Software 5.5 Shimadzu, Kyoto, Japan). A 2.6 μm Kinetex C18 column (150 × 4.6 mm; Phenomenex, Bologna, Italy) was used for the separation at a flow-rate of 1.5 mL/min. The column was maintained at 40 °C. The separation was performed by means of a linear gradient elution. Eluents were: (A) 0.1% H_3_PO_4_; (B) acetonitrile. The gradient was as follows: from 6% B to 20% B in 18 min; from 20% B to 60% B in 7 min; from 60% B to 90% B in 19 min; 90% B for 10 min and then 6% B for 5 min. DAD analysis was carried out in the range of 200–600 nm.

Flavonol aglycones were identified using pure standards of quercetin and kaempferol; flavonol glucosides were identified using quercetin 3-O-glucoside and kaempferol 3-O-glucoside, while other flavonol derivatives were tentatively identified based on their UV spectra as kaempferol (λmax 266 nm, 348 nm) and quercetin (λmax 258 nm, 356 nm) derivatives. Quantification of flavonols was performed by a calibration curve built with quercetin 3-O-glucoside with the DAD set at 354 nm. Anthocyanins were identified using pure standards of delphinidin 3-O-glucoside, cyanidin 3-O-glucoside, petunidin 3-O-glucoside, peonidin 3-O-glucoside and malvidin 3-O-glucoside. Quantification of anthocyanins was performed by a calibration curve built with cyanidin 3-O-glucoside with the DAD set at 520 nm. Flavanols were identified using pure standards of catechin, epicatechin and procyanidin B1. Flavanols were quantified by a calibration curve built with catechin with the fluorimetric detector set at λex 230 and λem 320. Phenolic acids were identified using pure standards of ferulic acid, *p*-coumaric acid and synapic acid, while phenolic acid derivatives were identified based on their UV spectra as ferulic acid and synapic acid (λmax 220 nm or 240 nm, 328 nm) derivatives. Phenolic acids were quantified by a calibration curve built with ferulic acid with the DAD set at 354 nm. Results were expressed as milligrams of phenolic compound per liter for the GS extract and milligrams of phenolic compound per kilogram of dry weight for the beans.

### 2.8. Ferric Ion Reducing Antioxidant Power (FRAP) Assay 

The FRAP assay was performed on the GS extract and bean extracts, according to a procedure described previously [27]. Briefly, FRAP reagent was prepared by adding 25 mL of 300 mM acetate buffer, pH 3.6, 2.5 mL of 10 mM 2,4,6-tripyridyl-*s*-triazine in 40 mM HCl and 2.5 mL of 20 mM FeCl_3_. The reaction mixture contained 0.4 mL of GS or bean extracts opportunely diluted with methanol:water:HCl (70:30:0.1, *v*/*v*/*v*) and 3 mL of FRAP reagent. The increase in absorbance at 593 nm was evaluated after 4 min of incubation at 37 °C against a blank with no extract addition. For each extract, 2–4 dilutions were assessed in duplicate. A methanolic solution of FeSO_4_ · 7H_2_O was used for calibration. Results were expressed as millimoles of Fe(II) sulfate equivalents per kilogram of dry beans.

### 2.9. Statistical Analysis of Data 

Experimental data were analyzed by one-way ANOVA using the least significant difference (LSD) as a multiple range test, and by non-linear regression analyses using Statgraphics 5.1 (STCC Inc.; Rockville, MD, USA). Results are reported as average ± standard deviation (SD).

## 3. Results and Discussion

### 3.1. Kinetics of Hydration

**Figure 1:**
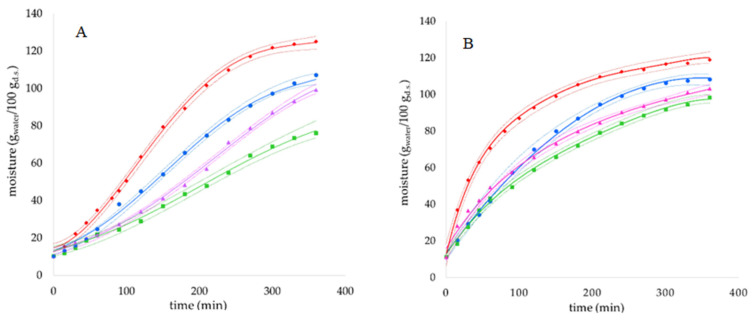
Hydration kinetics for the WKB (plot **A**) and CB (plot **B**) varieties. Symbols represent the average of experimental values, full lines represent the fitting f experimental data with the Kaptso et al. [24], (plot **A**) or the Peleg [23] (plot **B**) equations. Dotted lines represent 95% confidence limits. (♦), US-assisted soaking with water; (**●**), soaking with water; (**▲**), US-assisted soaking with GS extract; (■) soaking with GS extract.

The hydration kinetics of both *Phaseolus* species is shown in Figure 1 and Table 1. WKB showed a sigmoidal increase in water content with time, as observed for most species of the *Fabaceae* family [16], which could be modelled with the empirical equation by Kaptso et al. [23] with a good fit (R^2^ > 0.99). Conversely, CB showed a downward concave shape increase in water content with time, as observed for most species of the *Poaceae* family and some species of the *Fabaceae* family, which could be modelled with the empirical equation proposed by Peleg [24] (1988) with a good fit (R^2^ > 0.97).

The different hydration behavior among species of the *Fabaceae* family was attributed to differences in the size and shape of the *hilum*, through which the passage of water occurs [16]. The kinetic parameters observed for the control WKB hydrated with water, i.e., *τ* = 155 min, *k* = 0.013 min^−1^ were in the range of those reported for the same *Phaseolus* species (*τ* = 189 min and *k* = 0.011 min^−1^, [16]. For the control CB hydrated with water, the kinetic values observed, i.e., *k*1 = 1.18 min^−1^ · (g_water_/100 g_d.s._)^−1^, *k*2 = 0.0063 (g_water_/100 g_d.s._)^−1^ were in the range of those reported for pink kidney bean (*k*1 = 1.0 min^−1^ · (g_water_/100 g_d.s._)^−1^ and *k*2 = 0.0062 (g_water_/100 g_d.s._)^−1^, [16]). However, the RMSD and NRMDS values found in the current study were higher than those reported previously, probably due to the non-homogeneous size of the grains. 

The application of US during hydration with water of the WKB decreased the parameter *τ* by 20% and increased *k* by 30% and *M*_∞_ by 13%. Hence, both the initial and the final stage of the process were affected by US application. Interestingly, in the absence of US the WKB increased its absolute moisture content by 11.6-fold in 360 min, while the same increase occurred in 270 min upon application of US (Figure 1), suggesting a possible reduction of soaking time by 25% if US is applied. Previous studies have shown the acceleration effect of US on the sigmoidal hydration kinetics of other bean species, but the intensity of the resulting effect depends on the species and conditions applied [9,15]. 

For CB, the application of US during hydration with water decreased the parameter *k*1 by 50%, indicating a faster absorption of water in the initial phase of the process and increased *M*_∞_ by 8% (Table 1). A previous study has shown that US soaking of Navy beans, which exhibit a downward concave shape curve of hydration, significantly affects the *k*1 parameter but not *k*2, similar to the effect of increasing temperature [12]. As a result of fast kinetic of absorption, the increase in the absolute moisture content after 360 min was 9.7-fold without US, and the same increase occurred in 100 min only upon US application (Figure 1), suggesting a possible reduction of soaking time of 72%.

When hydration was performed with the GS extract, the kinetic of absorption and the equilibrium moisture content attained were slower with respect to those occurring with pure water, for both *Phaseolus* species. These effects can be attributed to the acid pH of the phenolic extract (3.5), which influences charge density of protein and pectic molecules, and ultimately the degree of hydration of beans. Indeed, the hydration kinetics of Faba bean was studied in the pH range 3–9, and it was observed that the process is faster under alkaline conditions [28]. Nevertheless, the application of US increased the amount of GS extract absorbed (*M*_∞_) in the *Phaseolus* specie that has a sigmoidal hydration behavior (WKB) by 28%. However, US application did not affect water absorption for the *Phaseolus* specie that has a downward concave shape hydration behavior (CK) (Figure 1, Table 1).

### 3.2. Antioxidant Content

Antioxidant content of WKB and CB is shown in Table 2 and Table 3, respectively.

#### 3.2.1. Flavonols

Dry WKB did not contain flavonols, as already observed [29]. Dry CB was found to contain 341 mg/kg_d.w._ of flavonols, including quercetin and kaempferol aglycones and their glycosides (Table 3). Soaking with water caused a remarkable decrease in flavonol glycosides in CB, due to leaching of these hydrophilic compounds into water; the corresponding aglycone kaempferol, which is less polar than the glycosides, increased with respect to the amount found in dry beans. The increase in flavonol aglycones during soaking was also observed in black kidney bean, and was attributed to the enzymatic activity of glycosidases [30]. In GS extract, quercetin 3-O-glucoside (17.0 ± 0.1 mg/L), quercetin (2.8 ± 0.4 mg/L), kaempferol 3-O-glucoside (4.1 ± 0.2 mg/L), and kaempferol (0.30 ± 0.01 mg/L) were identified. Based on the amount of GS extract absorbed (approximately 1 mL/kg d.s.), in both WKB and CB, flavonol aglycones were found in higher amount, while quercetin and kaempferol glycosides were recovered in lower amount with respect to those expected, probably due to glycosidase action as for the control beans soaked in water. In both varieties, US application resulted in higher recovery of total flavonols after soaking with GS. Heating caused a remarkable decrease in flavonol in both WKB and CB, as observed for other legumes [31]. After heating, US-soaked beans had a higher flavonol content than the control beans soaked in GS extract and beans soaked in water.

#### 3.2.2. Flavanols

Dry WKB was found to contain 10.9 mg/kg_d.w._ of flavanols, while dry CB contained 334 mg/kg_d.w._ of flavanols, among which catechin, epicatechin and procyanidin B1 (in CB only) were identified (Table 2 and Table 3). The flavanol content in dry beans is dependent on the species. Comparing six *P. vulgaris* varieties, catechin was generally found to be the prevalent flavanol monomer at 0–200 mg/kg and procyanidin B1 was found in the range 7–41 mg/kg. The monomer epicatechin was found only in some bean varieties [32]. In both WKB and CB, soaking with water decreased the content of these polar phenolics due to leaching. In GS extract, the amount of catechin was 8.3 ± 0.1 mg/L, epicatechin was 4.6 ± 0.1 mg/L and the dimer procyanidin B1 was 3.0 ± 0.3 mg/L. Based on these levels and the amount of GS extract absorbed (approximately 1 mL/kg d.s.), in both WKB and CB, the monomeric flavanols catechin and epicatechin in GS-soaked beans were found in higher amounts with respect to those expected, which could be due to the hydrolysis of flavanol oligomers/polymers occurring at the acidic pH of the GS extract. Indeed, the flavanol dimer procyanidin B1 did not increased in the GS-soaked beans with respect to water-soaked beans (Table 3). US application did not affect the amount of flavanols in the GS-soaked beans. Heating caused an increase in flavanol monomers both for water-soaked and GS-soaked beans, probably due to further hydrolysis of flavanol oligomers/polymers. Similarly, catechin was observed in higher concentration in cooked than in raw legumes of different species [29,33]. After heating, GS-soaked beans had higher flavanol content than water-soaked beans, but no significant effect of US was observed on flavanol content.

#### 3.2.3. Anthocyanins

As expected, anthocyanins were lacking in dry WKB (Table 2) while in dry CB, the amount of anthocyanin was 32.1 mg/kg_d.w._, including cyanidin 3-O-glucoside and petunidin 3-O-glucoside (Table 3). In common *P. vulgaris* varieties, anthocyanin profile and content depend on the variety and involves: petunidin 3-O-glucoside, cyanidin 3-O-glucoside and malvidin 3-O-glucoside [34], as well as delphinidin di-glucosides and cyanidin [32,35]. Soaking in water led to a complete loss of these polar compounds. Anthocyanins found in GS extract included delphinidin glucoside (19.1 ± 0.2 mg/L), cyanidin glucoside (5.6 ± 0.1 mg/L), peonidin glucoside (20.8 ± 0.1 mg/L), petunidin glucoside (5.9 ± 0.1 mg/L) and malvidin glucoside (47.2 ± 0.3 mg/L). Upon GS-soaking, only malvidin glucoside, delphinidin glucoside and petunidin glucoside were found in beans, although in lower amounts with respect to that expected. This result could be due to oxidation or condensation of these compounds during soaking, since their oxygen sensitivity is higher that oxygen sensitivity of other grape phenolics such as flavanols and flavonols [36]. Moreover, after heating only malvidin glucoside was observed in CB, suggesting that further degradation occurred.

#### 3.2.4. Phenolic Acids

In dry WKB and CB, a total of 524 and 673 mg/kg_d.w._ of phenolic acid derivatives were found (Table 2 and Table 3). In different *Phaseouls* species, phenolic acids were found to be the main phenolic class, occurring as free, conjugated to soluble oligosaccharides and peptides through hydrophobic and covalent ester and ether bonds or bounded to polysaccharides of the cell wall via ester linkage. The major phenolic acids are ferulic acid, *p*-coumaric acid and synapic acid [32,37]. In both WKB and CB, free phenolic acids were identified along with conjugated soluble forms. Soaking in water caused a decrease in these compounds, especially in CB. Instead, soaking with GS without US application caused a remarkable increase in ferulic acid. Since this compound was not present in GS, its increase could be due to the acidic pH of GS extract that favored its release from the cell wall. On the other hand, in both varieties, US-assisted soaking with GS led to lower phenolic acid content in the soaked beans than in the beans soaked in GS without US application, especially ferulic acid and the soluble conjugated forms. This result can be due to the increased diffusion in the soaking solution of these hydroxycinnamic acids upon release from the cell wall. Indeed, US facilitates the extraction of phenolic acids from *Phaseouls* [38]. After heat treatment, synapic acid content increased in all beans, but the other compounds decreased (Table 2 and Table 3). Previous studies have found that, upon heat treatment, phenolic acids can either increase [29] or decrease [33]. The overall trend depends on the prevalent effect of heat treatment among the release of phenolic acids from the cell wall, hydrolysis of the conjugated forms, and thermal degradation. In GS-soaked beans, heat treatment caused a decrease in these compounds, probably because most of their release had occurred in the previous soaking phase and hence the release during heating did not “balance” thermal degradation. Upon heat treatment, the highest levels of total hydroxycinnamic acid derivatives were found in the GS-soaked beans, as a result of greater retention of these compounds during soaking compared to US-soaking with GS or soaking in water.

### 3.3. FRAP Values

CB had much higher FRAP values than WKB, as expected from their phenolic profiles(Table 4). On the other hand, soaking with water did not affect FRAP values significantly in WKB but it significantly decreased FRAP values in CB, probably due to the loss of water-soluble phenolics with high antioxidant activity, such as anthocyanins and flavonols, as well as the remarkable loss in phenolic acids (Table 2 and Table 3). Accordingly, the effect of hydration on antioxidant activity of bean was found to be species-dependent [39].

Overall, WKB hydration with GS extract caused an increase in FRAP values from 1.16 to 2.07 mmol FeII eq/kg_d.w._ when US was applied and from 1.16 to 1.67 mmol FeII eq/kg_d.w._ in the control. Indeed, US application increased the amount of GS extract absorbed in WKB. In CB, soaking with GS extract also resulted in an increase in FRAP values from 5.3 to 7.5 mmol FeII eq/kg_d.w._, but the increase was not affected by US application, consistent with the equal amount of extract adsorbed. As discussed in the previous paragraph, hydration of beans with GS extract resulted in the absorption of anthocyanins, flavonols and flavanols. US application during GS soaking, was effective in increasing flavonol uptake, but it did not affect the uptake of anthocyanins and flavanols, which could be related to a higher polarity of these latter phenolic classes, which might have diffused in the soaking water. Moreover, US application resulted in a greater loss of the endogenous free and conjugated phenolic acids, probably due to an accelerating effect of their diffusion in the soaking water.

Heat-treated beans were not separated from the cooking water, hence the effects observed in the heated beans were only due to thermal degradation and not to leaching. The effects of heat treatment on the antioxidant activity of beans can be attributed to contrasting phenomena occurring in parallel. In particular, the degradation of thermolabile phenolic compounds such as anthocyanins, flavonols and free and conjugated phenolic acids, the hydrolysis of flavanol oligomers and polymers, increasing the levels of catechin and epicatechin and the release of matrix-bounded phenolics in free forms such as synapic acid.

As shown in Table 4, in WKB, US assisted soaking with GS extract prior to heating led to the highest FRAP value, which was 40% higher than that of the water-soaked control submitted to heat-treatment. In CB, soaking with GS extract without US application prior to heating led to the highest FRAP value, which was 53% higher than the water-soaked control submitted to heat treatment. 

The genetic diversity of common bean in phenolic quantity and composition and in the associated antioxidant activity has been a topic of extensive investigation [6]. Breeding strategies have been proposed to select the varieties that are rich in phenolic compounds, in order to maximize potential health benefits due to bean consumption, thus threatening the natural biodiversity [6]. In another perspective, the genetic diversity of common bean in seed permeability to water has also been considered, since this latter is an interesting trait to accelerate the soaking process [40]. However, seeds that exhibit a water-permeable tegument were found to undergo high phenolic loss during soaking [40]. Hence, the fortification with GS phenolics could be applied to enhance nutritional value of bean varieties with a naturally low phenolic content or to those varieties that are subjected to large phenolic losses during soaking. Moreover, there is an emerging interest in the use of beans to produce plant-based foods as meat alternatives [41,42]. It is worth noting that many foods belonging to the new generation of plant-based foods are assigned to ultra-processed foods that contain low levels of antioxidants and high levels of advanced glycation end products (AGEs), whose consumption has been linked to an array of chronic diseases [42]. Previous studies have shown that GS phenolics can inhibit the formation of AGEs [43]. Consequently, fortification with GS phenolics has also been recommended as one of the technological approaches to reduce the risks associated with AGEs formation in ultra-processed foods [41]. Hence, the fortification strategy proposed in this study could also be applied as a pre-treatment for bean intended to be submitted to intensive processing.

## 4. Conclusions

This study led to the following results: (a) US application can effectively decrease the soaking time of beans both in the model variety with a sigmoidal shape behavior and in the variety with a downward concave shape behavior during hydration; (b) US assisted soaking with GS was particularly effective for the uptake of GS flavonols by beans, but it did not affect the uptake of flavanols and anthocyanins with respect to the beans soaked in GS extract without US application; (c) US assisted soaking with GS extract decreased the endogenous free and conjugated phenolic acids with respect to soaking without US application. One limitation of this study is that the effects of US application during soaking are species-dependent and more advantageous for the bean variety with a low phenolic content. On the other hand, use of GS extract during soaking was successful as a fortification strategy for both species. These results can open-up novel strategies to innovate the soaking process in beans and ultimately the nutritional value of this food. Further studies should consider fortification strategies for value addition and differentiation of specific bean varieties and as a pre-treatment for the ultra-processed beans intended to provide a new generation of animal food analogues. 

## Figures and Tables

**Table 1:** Fitting of the hydration kinetics data using the Kaptso et al. [23] and the Peleg [24] models for the sigmoidal behavior of WKB and downward concave shape behavior of CB, respectively ^1^.

Sigmoidal Behavior–WKB				
	H_2_O-S	H_2_O-US-S	GS-S	GS-US-S
*τ* (min)	155 ± 5	123 ± 4	204 ± 25	217 ± 9
*k* (min^−1^)	0.013 ± 0.0006	0.017 ± 0.0008	0.009 ± 0.001	0.010 ± 0.0004
*M*_∞_ (g_water_/100 g_d.s._)	112 ± 3	127 ± 2	96 ± 9	123 ± 5
R^2^	0.997	0.996	0.985	0.998
RMSD	2.9	2.5	2.7	1.4
NRMSD	3.0	2.0	4.1	1.6
Downward concave shape behavior–CB				
	H_2_O-S	H_2_O-US-S	GS-S	GS-US-S
*k*1 (min · g_water_/100 g_d.s._)^−1^	1.18 ± 0.13	0.57 ± 0.07	1.47 ± 0.33	1.47 ± 0.13
*k*2 (g_water_/100 g_d.s._)^−1^	0.0063 ± 0.0004	0.0076 ± 0.0002	0.008 ± 0.001	0.0074 ± 0.0004
*M*_o_ (g_water_/100 g_d.s._)	8 ± 3	13 ± 3	17 ± 4.1	16 ± 2
*M*_∞_ (g_water_/100 g_d.s._)	113 ± 4	122 ± 4	97 ± 6	103 ± 3
R^2^	0.992	0.991	0.967	0.995
RMSD	2.9	2.9	4.9	2.0
NRMSD	3.0	2.7	5.6	2.2

^1^ H_2_O-S, soaking with water; H_2_O-US-S, US-assisted soaking with water; GS-S, soaking with GS extract; GS-US-S, US-assisted soaking with GS extract.

**Table 2:** Phenolic content (mg/kg_d.w._) of WKB at different processing steps ^1,2,3^.

		Soaked WKB			Soaked and Heated WKB		
	Dry WKB	H_2_O-S	GS-S	GS-US-S	H_2_O-S-H	GS-S-H	GS-US-H
Q-G	n.d.	n.d.	n.d.	n.d.	n.d.	n.d.	n.d.
Q	n.d.	n.d.	9.8 ^b^ ± 1	16.5 ^c^ ± 2	n.d.	2.7 ^a^ ± 0.3	4.7 ^a^ ± 0.3
K-G	n.d.	n.d.	n.d.	n.d.	n.d.	n.d.	n.d.
K	n.d.	n.d.	3.4 ^b^ ± 0.3	6.9 ^d^ ± 0.1	n.d.	2.0 ^a^ ± 0.1	5.1 ^c^ ± 0.1
Σ Flo			13.2 ^c^ ± 0.1	23.4 ^d^ ± 3.4		4.6 ^a^ ± 0.1	9.7 ^b^ ± 0.1
C	9.9 ^ab^ ± 1.9	5.4 ^a^ ± 0.1	14.7 ^bc^ ± 3.0	16.5 ^bcd^ ± 1.0	17.9 ^cd^ ± 1.1	23.2 ^de^ ± 0.5	26 ^e^ ± 6
E	1.0 ^a^ ± 0.1	0.53 ^a^ ± 0.1	1.8 ^ab^ ± 0.1	2.5 ^ab^ ± 0.1	1.5 ^a^ ± 0.1	4.2 ^ab^ ± 2.6	6.3 ^b^ ± 3.9
Σ Fla	10.9 ^ab^ ± 1.4	5.9 ^a^ ± 0.1	16.5 ^abc^ ± 3	19.0 ^bc^ ± 0.9	19 ^bc^ ± 3	27 ^cd^ ± 3	32 ^d^ ± 10
Mv-G	n.d.	n.d.	10.5 ± 2.6	7.6 ± 2.5	n.d.	n.d.	n.d.
Σ AC			10.5 ± 2.6	7.6 ± 2.5			
Fe	25.9 ^ab^ ± 0.1	28.6 ^b^ ± 0.5	39.7 ^c^ ± 1.7	16.9 ^a^ ± 1.6	19.0 ^a^ ± 0.5	19 ^a^ ± 6	24.5 ^ab^ ± 5
Sy	4.0 ^a^ ± 0.8	6.8 ^a^ ± 0.1	7.6 ^a^ ± 1.7	6.7 ^a^ ± 0.8	25.8 ^b^ ± 5.1	31.0 ^b^ ± 0.6	27.5 ^b^ ± 2.3
*p*-Cu	5.0 ^a^ ± 0.1	5.5 ^a^ ± 0.1	9.3 ^a^ ± 1.4	5.1 ^a^ ± 2.2	9.6 ^a^ ± 0.6	16 ^b^ ± 4	9.3 ^a^ ± 1.4
c HC	489 ^d^ ± 33	406 ^c^ ± 25	482 ^d^ ± 25	406 ^c^ ± 5	196 ^ab^ ± 7	236 ^b^ ± 8	161 ^a^ ± 7
Σ HC	524 ^d^ ± 33	447 ^c^ ± 26	539 ^d^ ± 20	435 ^c^ ± 1	250 ^a^ ± 14	302 ^b^ ± 10	222 ^a^ ± 31

^1^ H_2_O-S, soaking with water; GS-S, soaking with GS extract; GS-US-S, US-assisted soaking with GS extract; H_2_O-S-H soaking with water followed by heating; GS-S-H, soaking with GS extract followed by heating; GS-US-H, US-assisted soaking with GS extract followed by heating. ^2^ Different letters in a row indicate significant differences (*p* ≤ 0.05). ^3^ Q-G, quercetin glycosides; Q, quercetin; K-G, kaempferol glycosides; K, kaempferol; Σ Flo, sum of flavonols; C, catechin; E, epicatechin; Σ Fla, sum of flavanols; Mv-G, malvidin glucoside; Σ AC, sum of anthocyanins; Fe, ferulic acid; Sy, synapic acid; *p*-Cu, *p*-coumaric acid; c HC, conjugated hydroxycinnamic acids; Σ HC total hydroxycinnamic acid derivatives. n.d.; not observed.

**Table 3:** Phenolic content (mg/kg_d.w._) of CB at different processing steps ^1,2,3^.

		Soaked CB			Soaked and Heated CB		
	Dry CB	H_2_O-S	GS-S	GS-US-S	H_2_O-S-H	GS-S-H	GS-US-H
Q-G	115 ^b^ ± 3	n.d.	12.1 ^a^ ± 4.5	22.3 ^a^ ± 0.9	n.d.	n.d.	n.d.
Q	5.6 ^ab^ ± 1.6	1.6 ^a^ ± 1.1	13.5 ^d^ ± 2.7	20.0 ^e^ ± 1.5	n.d.	7.7 ^bc^ ± 0.2	10.2 ^cd^ ± 0.6
K-G	213 ± 1	n.d.			n.d.	n.d.	n.d.
K	7.2 ^b^ ± 0.1	14.1 ^d^ ± 2.0	9.8 ^c^ ± 1.0	17.6 ^e^ ± 0.3	2.9 ^a^ ± 1.1	8.2 ^bc^ ± 0.7	14.9 ^d^ ± 0.1
Σ Flo	341 ^f^ ± 28	15.7 ^b^ ± 0.9	35.4 ^d^ ± 3	59.9 ^e^ ± 0.3	2.9 ^a^ ± 1.1	15.9 ^b^ ± 0.6	25.1 ^c^ ± 0.3
C	210 ^cd^ ± 4	112 ^a^ ± 7	166 ^bc^ ± 38	161 ^b^ ± 31	146 ^ab^ ± 13	266 ^e^ ± 13	245 ^de^ ± 4
E	25 ^bc^ ± 4	6.9 ^a^ ± 0.1	12 ^ab^ ± 6	12 ^ab^ ± 1	35 ^cd^ ± 10	39 ^d^ ± 8	19 ^ab^ ± 2
P-B1	99 ^d^ ± 12	53 ^bc^ ± 5	56 ^bc^ ± 11	65 ^c^ ± 10	31 ^a^ ± 6	55 ^bc^ ± 11	43 ^ab^ ± 2
Σ Fla	334 ^c^ ± 12	172 ^a^ ± 3	234 ^a^ ± 55	238 ^ab^ ± 43	212 ^a^ ± 8	360 ^c^ ± 43	307 ^bc^ ± 6
Dp-G	n.d.	n.d.	3.5 ± 3.5	8.4 ± 1.9	n.d.	n.d.	n.d.
Cy-G	4.4 ± 1.6	n.d.	n.d.	n.d.	n.d.	n.d.	n.d.
Pt-G	27.7 ± 0.7	n.d.	8.2 ± 0.1	10.6 ± 0.5	n.d.	n.d.	n.d.
Pe-G	n.d.	n.d.	n.d.	n.d.	n.d.	n.d.	n.d.
Mv-G	n.d.	n.d.	27 ± 9.5	25.8 ± 9.5	n.d.	7.5 ± 0.6	7.9 ± 0.7
Σ AC	32.1 ± 0.9		38.7 ± 14	44.8 ± 10		7.5 ± 0.6	7.9 ± 0.7
Fe	60 ^b^ ± 10	31 ^a^ ± 5	127 ^c^ ± 21	59.5 ^b^ ± 8.7	31.1 ^a^ ± 0.1	74.6 ^b^ ± 4.9	27.2 ^a^ ± 1.7
Sy	11.1 ^b^ ± 2.4		9.1 ^ab^ ± 2	2.2 ^a^ ± 04	11.1 ^b^ ± 1.0	21.0 ^c^ ± 5.6	11.4 ^b^ ± 5
*p*-Cu	11.1 ^c^ ± 2.2		7.7 ^b^ ± 0.2	4.5 ^a^ ± 0.9	4.7 ^a^ ± 0.7	11.3 ^c^ ± 0.3	5.8 ^ab^ ± 0.4
cHC	591 ^d^ ± 8.4	100 ^a^ ± 4	417 ^c^ ± 75	150 ^ab^ ± 2	124 ^a^ ± 4	212 ^b^ ± 6	96 ^a^ ± 2
Σ HC	673 ^e^ ± 23	131 ^a^ ± 1	561 ^d^ ± 45	216 ^b^ ± 7	171 ^ab^ ± 2	319 ^c^ ± 17	140 ^a^ ± 4

^1^ H_2_O-S, soaking with water; GS-S, soaking with GS extract; GS-US-S, US-assisted soaking with GS extract; H_2_O-S-H soaking with water and heating; GS-S-H, soaking with GS extract and heating; GS-US-H, US-assisted soaking with GS extract and heating. ^2^ Q-G, quercetin glycosides; Q, quercetin; K-G, kaempferol glycosides; K, kaempferol; Σ Flo, sum of flavonols; C, catechin; E, epicatechin; P-B1, procyanidin B1; Σ Fla, sum of flavanols; DP-G, delphinidin glucoside; Cy-G, cyanidin glucoside; Pt-G, petunidin glucoside; Pe-G; peonidin glucoside; Mv-G, malvidin glucoside; Σ AC, sum of anthocyanins; Fe, ferulic acid; Sy, synapic acid; *p*-Cu, *p*-coumaric acid; c HC, conjugated hydroxycinnamic acids; Σ HC total hydroxycinnamic acid derivatives. n.d.; not observed. ^3^ Different letters in a row indicate significant differences (*p* ≤ 0.05).

**Table 4:** FRAP values (mmol FeII eq/kg_d.w._) of WKB and CB at different processing steps ^1,2^.

	Dry	Soaked			Soaked and Heated		
		H_2_O-S	GS-S	GS-US-S	H_2_O-S-H	GS-S-H	GS-US-H
WKB	1.23 ^a^ ± 0.13	1.16 ^a^ ± 0.17	1.67 ^b^ ± 0.27	2.07 ^c^ ± 0.30	1.21 ^a^ ± 0.22	1.46 ^ab^ ± 0.30	1.70 ^b^ ± 0.17
CB	9.3 ^e^ ± 0.4	5.3 ^c^ ± 1.3	7.5 ^d^ ± 1.5	7.5 ^d^ ± 1.1	2.8 ^a^ ± 0.4	4.3 ^bc^ ± 1.0	3.6 ^ab^ ± 0.2

^1^ H_2_O-S, soaking with water; GS-S, soaking with GS extract; GS-US-S, US-assisted soaking with GS extract; H_2_O-S-H soaking with water and heating; GS-S-H, soaking with GS extract and heating; GS-US-H, US-assisted soaking with GS extract and heating. ^2^ Different letters in a row indicate significant differences (*p* ≤ 0.05).

## Data Availability

Data is contained within the article.
